# Characteristics of Spatial Changes in Molars and Alveolar Bone Resorption among Patients with Loss of Mandibular First Molars: A CBCT-Based Morphometric Study

**DOI:** 10.3390/jcm12051932

**Published:** 2023-03-01

**Authors:** Huiyi Hong, Jing Zhou, Qi Fan, Ruijie Jiao, Qianyun Kuang, Hong Zhou, Chengge Hua, Zheng Yang, Wenli Lai, Hu Long

**Affiliations:** 1 State Key Laboratory of Oral Diseases, National Clinical Research Center for Oral Diseases, Department of Orthodontics, West China Hospital of Stomatology, Sichuan University, Chengdu 610041, China; 2 State Key Laboratory of Oral Diseases, National Clinical Research Center for Oral Diseases, Department of Pediatric Dentistry, West China Hospital of Stomatology, Sichuan University, Chengdu 610041, China; 3 West China School of Stomatology, Sichuan University, Chengdu 610041, China; 4 State Key Laboratory of Oral Diseases, National Clinical Research Center for Oral Diseases, Department of General Dentistry, West China Hospital of Stomatology, Sichuan University, Chengdu 610041, China

**Keywords:** cone beam computed tomography (CBCT), alveolar morphology, mandibular second molar

## Abstract

Objectives: To investigate the characteristics of spatial changes in molars and alveolar bone resorption among patients with loss of mandibular first molars. Methods: A total of 42 CBCT scans of patients with missing mandibular first molars (3 males, 33 females) and 42 CBCT scans of control subjects without loss of mandibular first molars (9 males, 27 females) were evaluated in this cross-sectional study. All images were standardized using the mandibular posterior tooth plane with Invivo software. The following indices regarding alveolar bone morphology were measured, including alveolar bone height, bone width, mesiodistal and buccolingual angulation of molars, overeruption of maxillary first molars, bone defects, and the capability of molar mesialization. Results: The vertical alveolar bone height in the missing group was reduced by 1.42 ± 0.70 mm, 1.31 ± 0.68, and 1.46 ± 0.85 mm on the buccal, middle, and lingual side, respectively (no differences among the three sides; *p* > 0.05). Alveolar bone width was reduced the greatest at the buccal CEJ level and the least at the lingual apex level. Mandibular second molar mesial tipping (with mean of the mesiodistal angulation = 57.47 ± 10.34°) and lingual tipping (with mean of the buccolingual angulation = 71.75 ± 8.34°) were observed. The mesial and distal cusps of maxillary first molars were extruded by 1.37 mm and 0.85 mm, respectively. Buccal and lingual defects of alveolar bone occurred at the CEJ, mid-root, and apex levels. Through 3D simulation, the second molar cannot be successfully mesialized into the missing tooth position, and the difference between the available and required distances for mesialization was the greatest at the CEJ level. The duration of tooth loss was significantly correlated with the mesio-distal angulation (R = −0.726, *p* < 0.001), buccal-lingual angulation (R = −0.528, *p* < 0.001) and the extrusion of the maxillary first molar (R = −0.334, *p* < 0.05). Conclusion: Both vertical and horizontal resorption of alveolar bone occurred. Mandibular second molars exhibit mesial and lingual tipping. Lingual root torque and uprighting of the second molars are needed for the success of molar protraction. Bone augmentation is indicated for severely resorbed alveolar bone.

## 1. Introduction

Clinically, loss of the mandibular first molar due to severe caries or periodontal disease is highly prevalent in adults. According to Almugla et al., there was a high prevalence of mandibular first molar loss (23.1% with one missing mandibular first molar, 13.3% with two, and 2.8% with three) in the general population in Saudi Arabia [1]. Similar results were observed in another study based on the population in Iran (17.05% with one missing mandibular molar, 10.4% with two, 7.2% with three, and 5% with four) [2]. Loss of mandibular first molars affects the masticatory function, aesthetics, structural balance, and psychological aspects of patients [3]. In order to solve these problems, restorations are commonly needed. The edentulous space can be restored by dental implants or bridge restorations [4,5,6,7]. However, dental implants and bridge restorations may not be the optimal option in some cases. Implants may fail due to broken necks, infection, or bone loss. According to the study of Levin et al., among 81 patients, mandibular first molar implants had a failure rate of 7.4%, and additional 11.1% of complications included suppuration and 2.5% included the presence of a pocket around the implant [8]. Additionally, most patients reject bridge restorations. Various concerns such as removable partial dentures and increased occlusal force of abutments are among the reasons for rejection. In addition, the downsides of bridge restoration include discomfort, loosening of prosthesis, and risks of root fracture and failure [9].

With the presence of the second and third molars, an alternative treatment plan is orthodontic protraction of the molars [5,10,11,12,13,14,15,16]. Natural teeth can be restored to the greatest extent. Third molars have similar crown sizes and root lengths compared to second molars. Therefore, second and third molars can substitute first and second molars to restore masticatory function. 

However, the success of molar protraction is one of the clinical challenges encountered by orthodontists. Compared to the maxillary complex, the mandible has thicker cortical bone, and the molar roots are much wider buccolingually [11,12,14,15]. Moreover, the overeruption of maxillary molars, alveolar bone loss, tipping of the adjacent teeth, a long mesialization distance, and strong bone resistance are the main challenges of a successful mandibular molar protraction [12,13,15]. As a result, mandibular molars are difficult to mesialize into the edentulous space. In previous case reports, some researchers have described successful mesialization of second molars into the edentulous space. However, some have reported the failure of molar protractions [10,11,12,14,15,16]. The dentoalveolar changes after the loss of first molars lead to the failure of molar protraction. The main reasons for mandibular first molar loss are due to severe caries and periodontal problems which usually result in severe alveolar bone loss after tooth extraction.

Previous studies focused on the alveolar morphology changes in the missing molar region and the unopposed maxillary first molar [17,18,19,20]. Craddock et al. found overeruption of maxillary first molars after the loss of opposing mandibular first molars [21]. Other researchers observed dentoalveolar changes in unilateral extractions of mandibular first molars [22,23,24]. Baik et al. found that mandibular second molars exhibited alveolar bone resorption after orthodontic movement of the second molars into the edentulous space using two-dimensional panoramic radiographs [25]. The change in bone morphology limits the protraction of second molars into the edentulous region, resulting in bone fenestration and molar tipping. To date, few studies have focused on the investigation of limitations of molar mesialization. In this study, we measured the mandibular posterior tooth tipping, bone loss, and maxillary molar overeruption and evaluated mesialization limitations using CBCT.

The objective of this study was to investigate the bone morphology change after the loss of the mandibular first molar, offering clinical clues for the protraction of mandibular molars.

## 2. Materials and Methods

This retrospective study used cone beam computed tomography (CBCT) images to assess the bone morphology changes in missing molar regions, further exploring the limitations of second molar protraction. The protocol was approved by the Ethical Committee of West China Hospital of Stomatology, Sichuan University. CBCT scans of patients with missing mandibular first molars were obtained from the Department of Oral Radiology, West China Hospital of Stomatology, Sichuan University.

For the missing molar group, patients between 18 and 40 years old were included if they presented with the maxillary first molar, mandibular first and second premolars, and mandibular second and third molars. Years elapsed between the loss of teeth and the performance of CBCT were determined and presented in months. The exclusion criteria were as follows: (1) history of any orthodontic treatment or orthognathic surgery, (2) craniofacial deformities or evident facial and skeletal asymmetry, and (3) generalized periodontitis. 

CBCT images were analyzed and divided into two groups: the missing group (with first molar missing) and the control group (with first molar present). For the missing group, as displayed in Table 1, 36 patients (28.7 ± 6.4 years) with 42 missing mandibular first molars were included in the study, with 13 patients missing the mandibular right first molar, 17 missing the mandibular left first molar, and 6 missing both mandibular first molars. The mean duration of tooth loss in the missing group was 62.6 ± 13.3 months. For the control group, 36 patients (26.1 ± 5.8 years) with 42 mandibular first molars were included in the study (Table 1).

All cases of loss of mandibular first molars were screened by an experienced radiologist and then reviewed by an experienced orthodontist. The CBCT scans of patients were exported in DICOM format and measured bilaterally using In Vivo Dental Application Version 5.1.6. Student’s t test and ANOVA were used to compare continuous variables. Pearson’s correlation analysis was used to evaluate the time effect. All tests were two-sided and a *p*-value < 0.05 was considered statistically significant. All statistical analyses were performed using SPSS Statistics Version 22.0.0.0. 

### 2.1. Determination of Coordinate Planes

**Figure 1:**
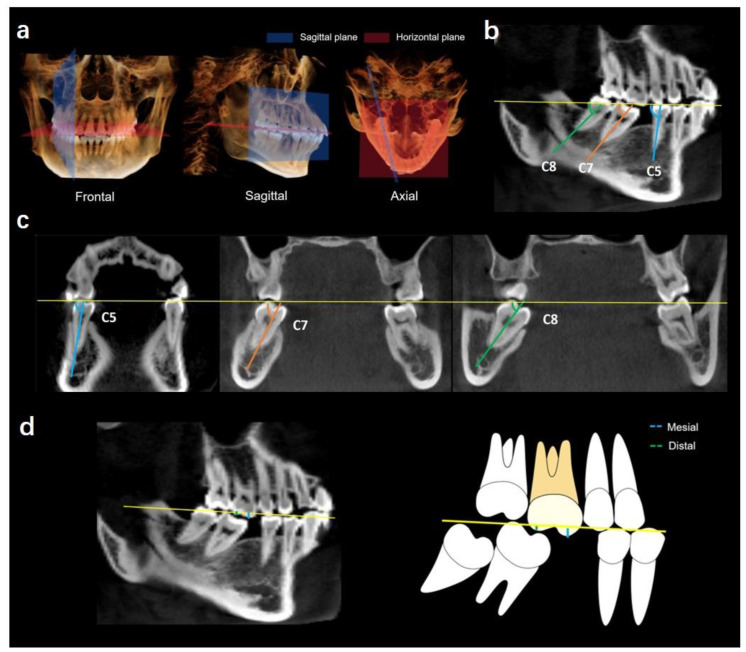
(**a**) Three-dimensional view of the coordinate plane. (**b**) Mesiodistal angulation of C5, C7, C8. The angles were measured in relation to the posterior tooth reference line. (**c**) Buccolingual angulation of C5, C7, C8. The angles were measured in relation to the posterior tooth reference line. (**d**) Overeruption of the maxillary first molar. The extent of extrusion was measured from the coordinate line (in yellow) to the tips of the mesial and distal cusps. The distances between the reference line and the molar cusps (the mesial cusp in blue and the distal cusp in green) were measured.

In the study, the mandibular posterior tooth plane was used as the reference plane to calibrate the CBCT scans for all measurements (Figure 1a). The mandibular posterior tooth plane was determined as the plane passing through the line connecting the cusps of posterior teeth at two sides. Once the reference plane was confirmed, the CBCT scans were calibrated as follows.

First, each scan was standardized and oriented in frontal view to make sure that the horizontal plane was parallel to the posterior occlusal plane. Then, the sagittal plane was defined as the plane passing through the long axis of the premolar and second molar in sagittal view. Finally, the coronal cross-section was defined as perpendicular to the posterior alveolar arch of the missing first molar side.

### 2.2. Measurements

#### 2.2.1. Angulations of Bilateral Mandibular Posterior Teeth

##### Mesiodistal Angulation

The mesiodistal angulation of the mandibular second molar was defined as the lower posterior angle between the long axis of the tooth and the posterior occlusal plane in the sagittal plane (Figure 1b). The long axis of the premolar (C5) was defined as the line passing through the mid-point of the crown and the root apex. The long axis of the molars (C7, C8) was defined as the line passing through the central fossa of the molar crown and the root furcation for multiple roots or the root apex for a single root. A smaller angulation degree indicates that the tooth tipped more mesially.

##### Buccolingual Angulation 

The buccolingual angulation of the tooth was defined as the angle between the long axis of the tooth and the posterior plane in the coronal section (Figure 1c). The long axis of the premolar was defined as the line passing through the mid-point of the crown and the root apex. The long axis of the molar was defined as the line passing through the central fossa of the molar crown and the root furcation or the root apex. A smaller angulation degree indicates that the tooth tipped more lingually.

#### 2.2.2. Extrusion of Maxillary First Molars

The extrusion of maxillary first molars was defined as the extrusion of the mesial cusp and distal cusp of the maxillary first molar. The amount of extrusion was measured from the posterior occlusal plane to the mesial and distal cusp (Figure 1d). 

#### 2.2.3. Alveolar Bone Loss in the Missing Tooth Region

##### Vertical Alveolar Bone Loss in Missing Tooth Region

**Figure 2:**
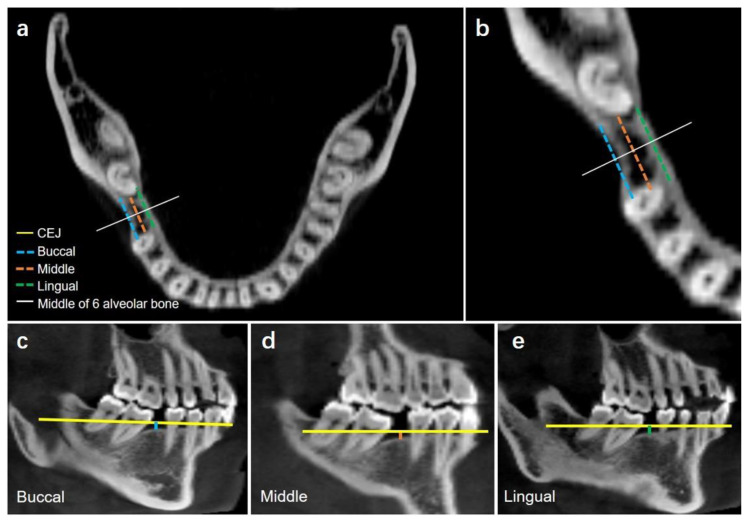
(**a**) Vertical alveolar bone levels of missing mandibular first molar. Coronal section showing the buccal, middle, and lingual sides of the missing first molar region. (**b**) Magnification. (**c**) Buccal vertical bone loss (mm): shortest distance (blue line) from the CEJ line (yellow line) to the alveolar crest in the middle of 6 alveolar bone region (white line). (**d**) Middle vertical bone loss (mm): shortest distance (orange line) from the CEJ line (yellow line) to the alveolar crest in the middle of 6 alveolar bone region (white line). (**e**) Lingual vertical bone loss (mm): shortest distance (green line) from the CEJ line (yellow line) to the alveolar crest in the middle of 6 alveolar bone region (white line).

In the missing tooth region, the vertical alveolar bone loss was defined as the vertical distance from the cemento-enamel junction (CEJ) to the alveolar bone crest of the adjacent teeth on the buccal, middle, and lingual sides respectively. (Figure 2). The buccal and lingual side of the alveolar crest were defined as the most buccal and lingual sides of the alveolar crest, respectively. 

##### Horizontal Alveolar Bone Loss in Missing Tooth Region

**Figure 3:**
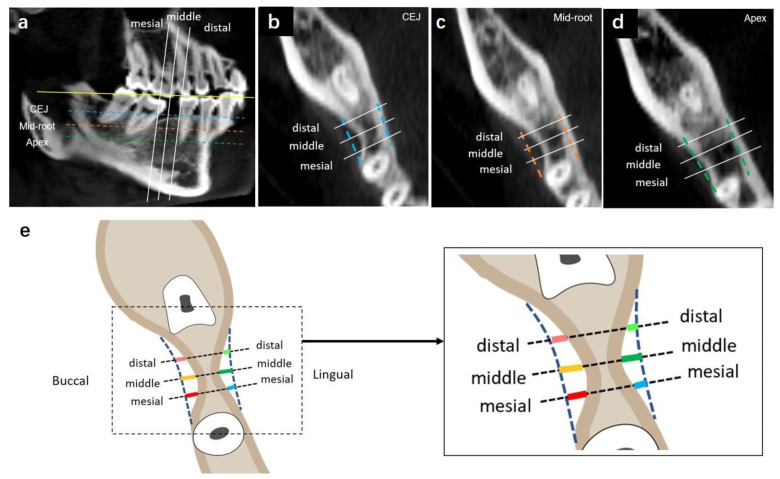
(**a**) Horizontal alveolar bone levels of the missing mandibular first molar. (**b**) Measurements at the CEJ level. (**c**) Measurements at the mid-root level. (**d**) Measurements at the apex level. (**e**) Illustration showing the measurements of 6 sites at three levels. A total of 6 sites (buccal: mesial, middle, distal; lingual: mesial, middle, distal) in each of the three sections (CEJ, mid-root, and apex) were taken. The line drawn perpendicular to the original bone cortex from the 6 sites was measured as the horizontal resorption of the alveolar bone.

The horizontal alveolar bone loss was defined as the differences between the original alveolar bone width and the actual alveolar bone width. First, the original bone width was established by drawing lines from the bone cortex of second molar to the bone cortex of the second premolar on both the buccal and lingual sides. Both buccal and lingual sides of the mesial and middle distal width in each coronal section (CEJ, mid-root, and apex) were measured (Figure 3). A total of 18 measurements were recorded.

#### 2.2.4. Evaluation of the Capability of Second Molar Protraction

##### Capability of Mesialization through Simulation of Molar Protraction

Both the required mesialization distance of the second molar and the available mesialization distance of the second molar were measured based on the 3D simulation of second-molar protraction. The required and available mesialization distances of second molars were measured at the CEJ, mid-root, and apex levels.

- The required mesialization distances of second molars

**Figure 4:**
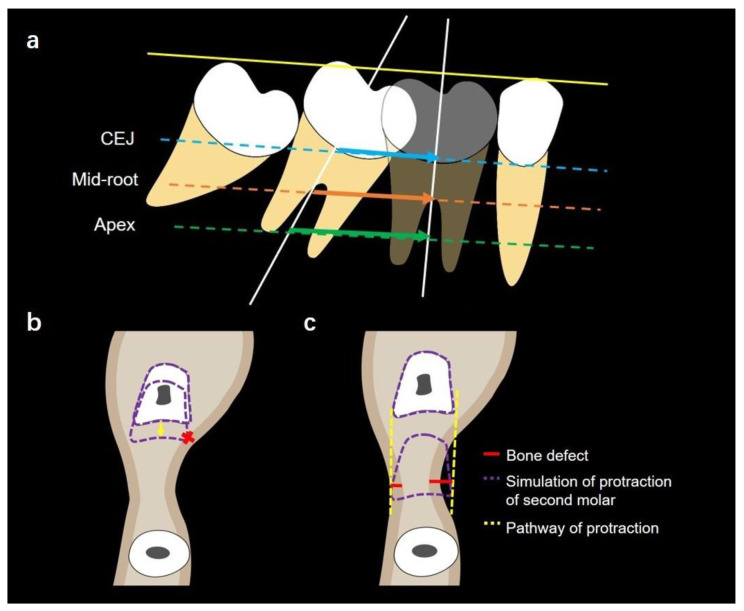
(**a**) Illustration showing the required mesialization distances of second molars. The second molar was mesialized to the position of the missing first molar through simulation. The line drawn from the most mesial mid-point of the original second molar and the mesialized second molar at CEJ (shown in blue arrow), mid-root (shown in orange arrow), and apex levels (shown in green arrow) was measured. (**b**) Illustration showing the available mesialization distances of second molars. The shortest distances (yellow line) from the most mesial mid-point of the original second molar to the mesialized second molar at CEJ, mid-root, and apex levels were measured when the second molar contacted the cortical bone through mesialization. (**c**) Illustration showing buccal and lingual bone defects of second molar protraction through 3D simulation. The line (shown in red line) perpendicular to the pathway of the second molar protraction (yellow dotted line) was drawn from the most concave inner layer of the bone cortex to the pathway of the second molar protraction on the buccal and lingual sides. Buccal and lingual bone defects (red lines) were measured.

The required mesialization distance of second molars was defined as the distance of the second molar traveled to replace the missing first molar. The second molar (shown in arrows in Figure 4a) was virtually moved to the position of the missing first molar through 3D simulation. The lines drawn from the most mesial mid-point of the original second molar and the mesialized second molar at CEJ (shown in blue arrow), mid-root (shown in orange arrow), and apex levels (shown in green arrow) were measured as the required mesialization distance of second molars.

- The available mesialization distances of second molars

In some cases where there was alveolar bone resorption, the available mesialization distances of second molars were measured (Figure 4b). If any alveolar bone resorption was presented, the available mesialization distance of the second molar was defined as the shortest distance from the most mesial mid-point of the original second molar to the virtually-mesialized second molar when the second molar contacted the cortical bone through mesialization. The narrowed alveolar bone width indicated bone defects. 

##### Bone Defects of Second Molar Protraction through 3D Simulation

Bone defects were defined as the width differences between the second molar and alveolar bone in the edentulous region. The pathway of the second molar protraction was determined through 3D simulation and shown by the yellow dotted line in Figure 4c. The second molar was outlined by the purple dotted line in order to show the virtual protraction. When the second molar was mesialized to the position of the missing first molar, a line (shown in red line) perpendicular to the pathway of the second molar protraction was drawn from the most concave inner layer of the bone cortex to the pathway of the second molar protraction on the buccal and lingual sides. Both buccal and lingual bone defects at CEJ, mid-root, and apex levels were measured.

## 3. Results

### 3.1. Tipping of Mandibular Posterior Teeth

**Figure 5:**
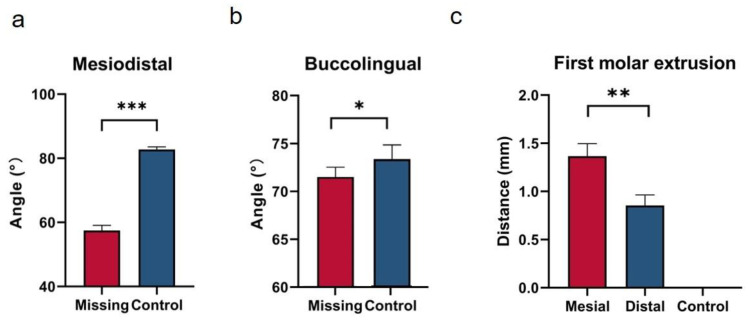
(**a**) Mesiodistal angulation of the mandibular second molar in missing group and control group. (**b**) Buccolingual angulation of the mandibular second molar in missing group and control group. (**c**) Extrusion of the maxillary first molar in missing group and control group. * indicates *p* < 0.05, ** indicates *p* < 0.01, *** indicates *p* < 0.001.

In the mesio-distal dimension, our results revealed that posterior teeth in the missing group tended to tip toward the edentulous region compared with the control group, as shown in Figure 5a (missing group: 57.47 ± 10.34°, control group: 82.75 ± 5.14°). Particularly, second molars exhibited significant mesial tipping in the missing group compared to the control group (*p* < 0.001). 

In the bucco-lingual dimension, second molars had significantly more lingual tipping in the missing group when compared with those in the control group (missing group: 71.75 ± 8.34°, control group: 73.23 ± 7.60°) (*p* < 0.05), as shown in Figure 5b. 

### 3.2. The Extrusion of Maxillary First Molars

As illustrated in Figure 5c, the overeruption of first molars was only found in the missing group (mesial cusp: 1.37 ± 0.83 mm, distal cusp: 0.85 ± 0.70 mm). Particularly, a significant difference was observed between the mesial cusp and distal cusp (*p* < 0.01).

### 3.3. Alveolar Bone Loss in Missing Tooth Region

**Figure 6:**
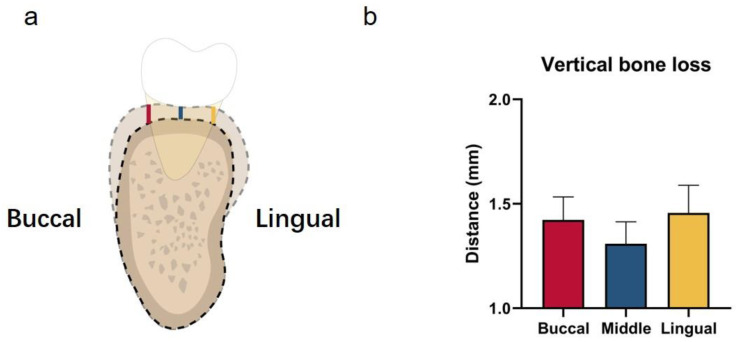
(**a**) Illustration showing the measurements of vertical alveolar bone loss on buccal, middle, and lingual sides. (**b**) Vertical bone loss on buccal, middle, and lingual sides.

We found that the vertical alveolar bone height on the buccal, middle, and lingual sides decreased in the missing group (Figure 6). However, no significant differences were observed among these three sides (*p* = 0.64).

**Figure 7:**
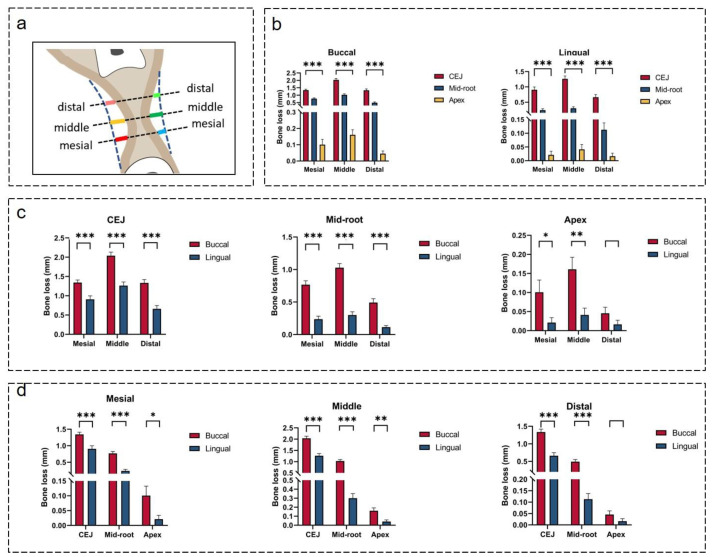
(**a**) Illustration showing horizontal alveolar bone loss. (**b**) Comparisons of horizontal bone loss at different sites. (**c**) Comparisons of buccal and lingual bone loss at mesial, middle, and distal sites. (**d**) Comparisons of buccal and lingual bone loss at CEJ, mid-root, and apex levels. * indicates *p* < 0.05, ** indicates *p* < 0.01, *** indicates *p* < 0.001.

As displayed in Figure 7b and Table 2, the horizontal alveolar bone width decreased at the CEJ, mid-root, and apex levels (CEJ > mid-root > apex). In particular, bone resorption on the buccal side was greater than that on the lingual side (Figure 7c,d).

### 3.4. Evaluation of the Capability of Second Molar Protraction 

**Figure 8:**
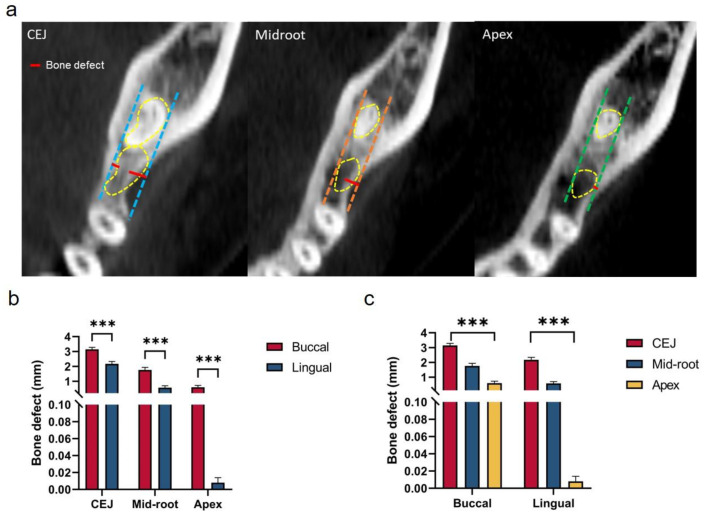
(**a**) Measurements of buccal and lingual bone defects at CEJ, mid-root, and apex levels. (**b**) Comparisons of the buccal and lingual bone defects. (**c**) Comparisons of the bone defects at CEJ, mid-root, and apex levels. *** indicates *p* < 0.001.

Our results indicated inadequate buccal and lingual alveolar bone at CEJ, mid-root, and apex levels. As shown in Figure 8b, buccal bone defects were larger than those on the lingual side at all the three vertical levels (CEJ, mid-root and apex levels). Bone defects at the CEJ level were the largest while bone defects at the apex level were the smallest, as shown in Figure 8c (buccal defect of alveolar bone at CEJ level: 3.15 ± 0.87 mm, lingual defects of alveolar bone at apex level: 0.01 ± 0.04 mm).

**Figure 9:**
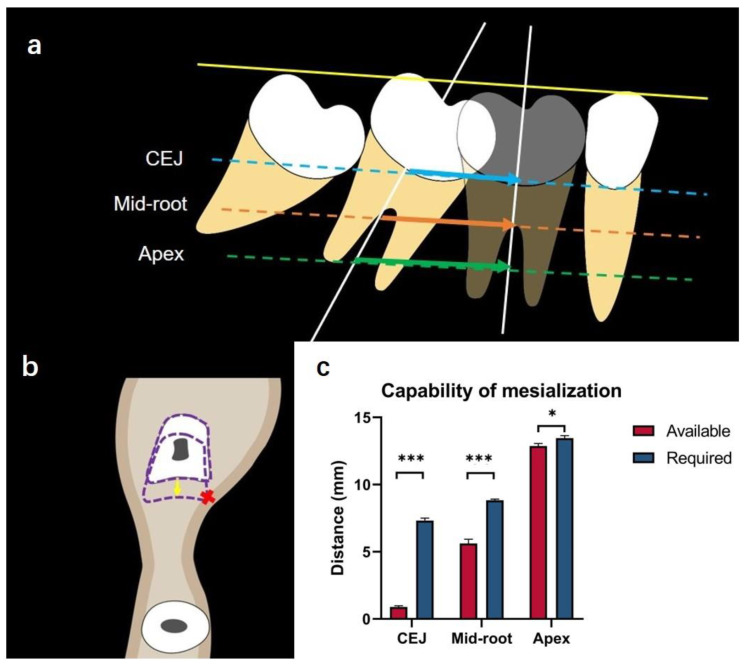
(**a**) Illustration showing the measurements of required mesialization distances. (**b**) Illustration showing the measurements of available mesialization distances. (**c**) Available and required mesialization distances at CEJ, mid-root, and apex levels. * indicates *p* < 0.05, *** indicates *p* < 0.001.

The available distances of second molar mesialization were smaller than the required distance at all the three vertical levels (CEJ, middle, and apex levels) (Figure 9 and Table 3). Significant differences were observed between the available mesialization distance and the required mesialization distance at CEJ (*p* < 0.001), middle (*p* < 0.001), and apex levels (*p* < 0.05). 

### 3.5. Evaluation of the Correlation of Parameters and the Duration of Tooth Loss

As shown in Table 4, our results indicated that the duration of tooth loss was correlated strongly with the mesio-distal angulation (R = −0.726; *p* < 0.001). Additionally, the results of correlation analysis demonstrated that the duration of tooth loss was correlated with buccal–lingual angulation (R = −0.528; *p* < 0.001) and the extrusion of the first molar (R = −0.334; *p* < 0.05).

## 4. Discussion

In our study, mandibular second molars exhibited mesial tipping in the missing group with an average mesio-distal inclination of 57.47 ± 10.34°. A similar trend was reported in previous studies [22]. This indicates that the second molar tends to mesially tip towards the edentulous space. The tendency of lingual tipping of second molars was also observed in this study. In our study, mandibular second molars were lingually tipped in the missing group with an average bucco-lingual inclination of 71.75 ± 8.34°. As shown in Table 4, the duration of tooth loss was correlated with the mesio-distal angulation, buccal–lingual angulation, and the extrusion of the maxillary first molar. This indicates that greater mesial and lingual tipping of mandibular second molars may occur in patients with longer durations of mandibular first molar loss, justifying a timely treatment for the loss of mandibular first molars. However, the mesio-distal angulation of the second molar was more stronger correlated with the duration of tooth loss than the buccal-lingual angulation. This is probably due to the thick lingual cortical bone and the occlusion with the upper teeth that prevented the lingual tipping of the second molar. 

Our study found that alveolar bone resorption was significantly greater on the buccal side than that on the lingual side. This is probably due to the greater cortical bone thickness on the lingual side compared to that on the buccal side. In addition, after a long period of the loss of the mandibular first molar, the buccal cortical plate collapses and impedes the tooth movement. In order to mesialize molars successfully, corticotomy is indicated to reduce bone resistance and to accelerate molar protraction. Moreover, molar protraction demands high requirements on anchorage and may lead anterior anchorage loss that is manifested as lingual tipping of anterior teeth, especially mandibular incisors. Thus, to preserve anchorage in the anterior teeth, mini-implants, as an absolute anchorage modality, are clinically indicated. Both direct and indirect anchorage modes can be employed for mini-implants. For direct anchorage, a protraction loop can be fabricated and attached to the mini-implant and the second molar with a stainless wire in order to provide a protraction force with an upright moment and lingual root torque to the second molar. For indirect anchorage, a stainless wire can be fabricated and attached to the canine or premolar in order to prevent them from distal tipping.

Another point of discussion may be related to the alveolar bone resorption. Our study showed both vertical and horizontal alveolar bone loss in the missing tooth region. Recent studies also reported similar results [10,25]. Periodontal disease, age, and oral hygiene are associated with bone resorption. Moreover, through the simulation of molar protraction, we found that inadequate buccal and lingual alveolar bone were present. Bone dehiscence and fenestration may occur during molar protraction. Therefore, alveolar bone grafting and augmentation may be necessary. Patients may undergo alveolar ridge splitting and bone graft [26,27]. Recent findings supported that vertical and horizontal bone loss caused by alveolar bone resorption due to missing mandibular first molars are indications of alveolar ridge splitting [28,29]. The buccal cortical plate collapses and obstructs the protraction pathway of molars. Alveolar ridge splitting can reconstruct the morphology of the alveolar crest and reduce the resistance from the cortical plate during molar protraction [30]. Alveolar bone grafting includes ridge preservation and ridge augmentation [31]. Bone graft can increase the amount of bone and promote bone formation, supporting the long-term stability of molars [32]. However, there is still no single grafting material recommended as the gold-standard grafting material. All grafting materials show advantages and downsides. In addition, long surgery treatment time, graft failure, and soft tissue complications may be concerns for surgery [26,28].

According to the extrusion of the maxillary first molar (Figure 5), the maxillary first molar over-erupted in the missing group. This was mainly caused by no opposing tooth for the maxillary first molar in the edentulous space of the mandible. Without occlusal contact, unopposed maxillary first molars over-erupted, which was consistent with previous findings [17,21]. Christou et al. and Craddock et al. suggested that there was vertical displacement of unopposed molars [17,21]. Therefore, intrusion of maxillary first molars is needed.

Based on the significant differences observed between the available mesialization distance and the required mesialization distance (Figure 7 and Table 2), orthodontic protraction of mandibular second molars may be hindered by the collapsed buccal cortical plate. Thus, lingual root torque and uprighting of the second molar are indicated for the success of molar protraction. Several available approaches for molar uprighting, such as Niti coil spring, cantilever spring, and helical uprighting spring, are commonly used for orthodontic treatments [33,34,35]. However, the mandible has thick cortical bone and second molar roots are usually wide in the buccolingual dimension. Therefore, a strong anchorage control is needed for molar uprighting [36]. A mandible full-arch fixed appliance with stainless-steel wire or mini-implant is suggested for molar uprighting [37]. Molar uprighting using mini-implants can shorten the treatment time, and serve as a direct or indirect anchorage control [38,39]. Mini-implants can minimize the side effects caused by molar uprighting, such as undesired movement of anchorage teeth units and long treatment time [40,41]. It has been suggested that molar uprighting requires sufficient anchorage control, and with the use of orthodontic mini-implants, it shows a satisfactory treatment outcome for molar uprighting [42]. 

## 5. Conclusions

**Figure 10:**
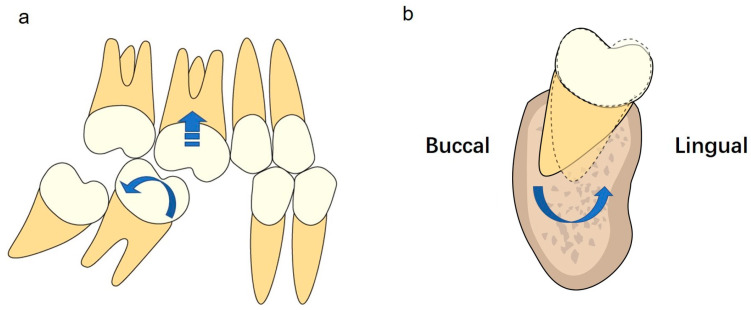
(**a**) Uprighting of second mandibular molars and intrusion of maxillary first molars are recommended for the protraction of mandibular second molars. (**b**) Lingual root torque of second mandibular molars is recommended for the protraction of mandibular second molars.

1. Mesial and lingual tipping of mandibular second molars and extrusion of maxillary first molars may occur among patients with missing mandibular first molars.
2. Alveolar bone resorption is exhibited in both the vertical and horizontal dimensions following the loss of mandibular first molars.
3. Alveolar bone resorption is greater on the buccal side than on the lingual side.
4. The quantity of the buccal bone at the CEJ level is the limiting factor in determining the capability of second molar protraction, and alveolar bone augmentation may be indicated for molar protraction.
5. Molar uprighting and lingual root torque of mandibular second molars and intrusion of maxillary first molars are recommended for the protraction of mandibular second molars (Figure 10).

## Figures and Tables

**Table 1:** Patient characteristics.

	Missing Group (N = 36)	Control Group (N = 36)	*p* Value
Age	28.7 ± 6.4	26.1 ± 5.8	*p* > 0.05
Sex			*p* > 0.05
Male	3	9	
Female	33	27	
Missing site			
Right	13	-	-
Left	17	-	-
Bilateral	6	-	-
Total count of missing teeth	42	-	-
Duration of tooth loss (months)	62.6 ± 13.3	-	-

Descriptive statistics (number) of demographic and clinical characteristics in the missing group and control group; Age data were presented as mean ± SD.

**Table 2:** Horizontal alveolar bone loss (mm) in missing group.

	Mesial		Middle		Distal	
	Buccal	Lingual	Buccal	Lingual	Buccal	Lingual
CEJ	1.34 ± 0.41	0.90 ± 0.58	2.04 ± 0.60	1.26 ± 0.60	1.33 ± 0.55	0.66 ± 0.55
Mid-root	0.77 ± 0.37	0.24 ± 0.30	1.03 ± 0.41	0.30 ± 0.33	0.49 ± 0.38	0.11 ± 0.16
Apex	0.10 ± 0.20	0.02 ± 0.08	0.16 ± 0.20	0.04 ± 0.11	0.05 ± 0.10	0.02 ± 0.07

**Table 3:** Capability of mesialization (mm) in missing group.

	Capability of Mesialization		*p* Value
	Available	Required	
CEJ	0.89 ± 0.57	7.32 ± 1.20	*p* < 0.001
Mid-root	5.62 ± 1.94	8.82 ± 0.61	*p* < 0.001
Apex	12.82 ± 1.06	13.89 ± 1.11	*p* < 0.05

**Table 4:** The correlation coefficients of each parameters and duration of tooth loss.

	Time (R)	*p* Value
Angulation		
Mesio-distal ***	−0.726	*p* < 0.001
Buccal–lingual ***	−0.528	*p* < 0.001
First molar extrusion		
Mesial cusp	−0.304	*p* > 0.05
Distal cusp *	−0.334	*p* < 0.05
Bone loss		
Buccal Middle	−0.024	*p* > 0.05
	0.034	*p* > 0.05
Lingual	−0016	*p* > 0.05

* indicates statistical significance with a *p* value less than 0.05, *** indicates statistical significance with a *p* value less than 0.001.

## Data Availability

The data presented in this study are available from the corresponding author upon reasonable request.
